# Correction to: ‘Gravitational waves from a first-order electroweak phase transition: a brief review’ (2018) by Weir

**DOI:** 10.1098/rsta.2023.0212

**Published:** 2023-10-16

**Authors:** David J. Weir

*Phil. Trans. R. Soc. A* **376**, 20170126. (Published online 6 March 2018) (https://dx.doi.org/10.1098/rsta.2017.0126).

## 1. Issues with the sound wave power spectrum formula

Equation (5.7) based on reference [59] unfortunately includes a number of errors. Firstly, in reference [59], the equivalent equation in the main body of the paper, equation (45), appears without the reduced Hubble constant squared $h^{2}$ on the left-hand side, where the present-day Hubble constant is defined as $H_{0}=h\times 100\;{\mathrm{km}\;s}^{-1}\;{\mathrm{Mpc}}^{-1}$.

The remainder of the issues are corrected in the Erratum to reference [59]:

— There was a factor of 3 missing from the right-hand side of equation (39), which yields a factor 3 in equation (45).
— The numerical coefficient in equation (45) should have been 0.687, not 0.68.
— The value quoted for ${\tilde{\Omega }}_{\text{gw}}$ in the main body was an order of magnitude too large and should be ${\tilde{\Omega }}_{\text{gw}}=1.2\times 10^{-2}$.

Incorporating these changes, and multiplying both sides by $h^{2}$, with the contemporaneous Planck best-fit value h=0.678 (also used in reference [59]), we arrive at the following replacement formula for this paper’s equation (5.7):

1.1 $$\begin{array}{r} h^{2}\Omega_{\text{sw}}(f)=1.19\times 10^{-6}{\left(\frac{100}{g_{*}}\right)}^{1/3}\Gamma^{2}{\bar{U}}_{f}^{4}\left(\frac{H_{*}}{\beta }\right)v_{w}\;S_{\text{sw}}(f), \end{array}$$

noting that $R_{*}={\left(8\pi \right)}^{1/3}v_{w}/\beta$ as in equation (3.3). The gravitational wave power spectrum shown in figure 6 is also therefore incorrect. An updated plot is, however, not included here for several reasons: our understanding of turbulence after a phase transition has improved since this paper came out; the expected LISA sensitivity curve has changed; and foregrounds are neglected.

As source modelling has improved since this paper was originally published, the interested reader is encouraged to compare the formulae in this paper with more recent results.

I apologise for any confusion caused.

